# Whole systems traditional Chinese medicine utilization among women undergoing frozen embryo transfers at an academic fertility center

**DOI:** 10.1016/j.xfre.2026.06.008

**Published:** 2026-07-03

**Authors:** Samuel N. Rodgers-Melnick, Lee Hullender Rubin, Sung Tae Kim, Lucas D. Jones, Rebecca Flyckt, Christine Kaiser

**Affiliations:** a Connor Whole Health, University Hospitals of Cleveland, Cleveland, Ohio; b Department of Psychiatry, Case Western Reserve University School of Medicine, Cleveland, Ohio; c Department of Population and Quantitative Health Sciences, Case Western Reserve University School of Medicine, Cleveland, Ohio; d Rosefinch Health LLC, Portland, Oregon; e Division of Reproductive Endocrinology and Infertility, University Hospitals Cleveland Medical Center, Cleveland, Ohio; f Department of Reproductive Biology, Case Western Reserve University School of Medicine, Cleveland, Ohio

**Keywords:** Whole Systems Traditional Chinese Medicine, acupuncture, health services research, in vitro fertilization, frozen embryo transfer

## Abstract

**Objective:**

To investigate sociodemographic and clinical variables associated with Whole Systems Traditional Chinese Medicine (WS-TCM) utilization alongside frozen embryo transfers (FETs) after in vitro fertilization.

**Design:**

We conducted a retrospective review of all autologous FETs performed within an academic medical center’s fertility clinic between January 2019 and March 2024. One FET cycle per patient was randomly selected for analysis. After conducting bivariate analyses, a logistic regression model was employed to examine independent associations with WS-TCM utilization.

**Subjects:**

Women aged 21–42 who underwent an FET after in vitro fertilization. FETs involving donor egg embryos, gestational carriers, and those with missing live birth outcome data were excluded.

**Exposure:**

Attending ≥1 WS-TCM appointment between 120 days pre- and 12 days post-FET. Whole Systems Traditional Chinese Medicine treatment modalities included individualized acupuncture, nutritional and lifestyle modifications, and focused dietary supplements.

**Main Outcome Measures:**

Sociodemographic and clinical variables associated with WS-TCM utilization.

**Results:**

Of 799 women studied, 162 (20.3%) utilized WS-TCM, receiving a median of 5.5 treatments between 120 days pre- and 12 days post-FET. Whole Systems Traditional Chinese Medicine users resided in neighborhoods with higher median income ($83,025 vs. $75,602). Previous medical histories of women incorporating WS-TCM included higher rates of migraine/headache (21.6% vs. 13.3%), musculoskeletal pain (37.0% vs. 27.5%), recurrent pregnancy loss (RPL; 25.9% vs. 12.9%), pain associated with genital organs (21.0% vs. 14.1%), and undergoing more assisted reproductive technology cycles. Higher neighborhood median income (adjusted odds ratio and 95% confidence interval: 1.36 [1.13, 1.64]), recurrent pregnancy loss (2.15 [1.33, 3.47]), and undergoing ≥3 vs. 0–1 assisted reproductive technology cycles (1.78 [1.02, 3.06]) were independently associated with higher odds of WS-TCM engagement, whereas higher body mass index (0.79 [0.64, 0.97]), undergoing preimplantation genetic testing (0.61 [0.39, 0.94]), and having a history of salpingitis/oophoritis/pelvic inflammatory disease (0.59 [0.37, 0.92]) or high-risk pregnancy supervision (0.53 [0.30, 0.93]) were associated with lower odds.

**Conclusion:**

Within this cohort, multiple pain- and reproductive health-related diagnoses were more prevalent among individuals utilizing WS-TCM. Findings inform factors that may influence clinicians’ and patients’ decisions to refer or pursue WS-TCM (e.g., migraine, musculoskeletal pain, RPL) and covariates that should be accounted for in future observational studies of WS-TCM’s effectiveness.

An estimated 10.43 million US women aged 15–49 are affected by infertility (1), of which 12.2% are estimated to have ever used infertility services (2) such as in vitro fertilization (IVF) (2). The Society for Assisted Reproductive Technology reports 415,953 IVF cycles were completed in 2023 (3), an increase from 395,641 cycles reported in the previous year (4). Since the first reported IVF live birth in 1978, approximately 9.8–13.0 million infants have been born worldwide through assisted reproductive technology (ART) (5).

People undergoing IVF seek options to manage the inherent stress of treatments and optimize their chances of pregnancy and live birth (6). They often engage in adjuvant integrative health and medicine (IHM) approaches like acupuncture or Whole Systems-Traditional Chinese Medicine (WS-TCM) to address these goals. Whole Systems Traditional Chinese Medicine is a multimodal intervention generally provided by licensed acupuncturists that can include acupuncture and its associated manual therapies, Chinese herbal medicine, lifestyle modifications, Chinese Medicine–based dietary recommendations, and nutritional supplement recommendations (7, 8, 9, 10). Several surveys report 8–47% of patients undergoing IVF add acupuncture (11, 12, 13, 14, 15). In the United States, WS-TCM treatment may be provided over a series of once- or twice-weekly sessions (8, 16).

Although IHM approaches like acupuncture and herbs are growing in the United States, acupuncture is mainly used for pain (17). In an analysis of the 2019 National Health Interview Survey, people with chronic pain who engaged in IHM were more likely to be female, reside in a metropolitan area, and have higher family income and education achievement than those not utilizing IHM (18). Older age, male sex, and Black or African American race were associated with lower odds of IHM utilization (18).

In trials, acupuncture is largely investigated as an adjuvant to embryo transfer (ET). Randomized controlled trials (RCTs) published in English predominantly examine acupuncture’s effects on fresh IVF cycle outcomes (19, 20, 21). Assisted reproductive technology practice in the United States has shifted toward frozen cycles over the last two decades. From 2006 to 2012, US patients completed 82.5% more frozen embryo transfer (FET) cycles, whereas fresh IVF-ET cycles increased only 3% (22). A meta-analysis of acupuncture’s impact on FET outcomes identified 14 RCTs with 1,130 participants for inclusion with all trials completed in China, of which only two studies were published in English (23).

To date, no RCTs completed in a US private or academic setting evaluating acupuncture alone or WS-TCM’s influence on IVF birth outcomes are published. In a retrospective study at a private US fertility clinic, WS-TCM (n = 119) was associated with (1) improved live birth rates (adjusted odds ratio [aOR] and 95% confidence interval [CI] 2.09 [1.36, 3.21]); and (2) lower biochemical pregnancy rates (0.28 [0.09, 0.91]) in fresh ETs compared with no treatment (n = 580) (8). In a second retrospective study at a Chinese academic affiliated hospital, WS-TCM (n = 356) was associated with an increase in FET live births (1.28 [1.01, 1.63]) compared with no treatment (n = 692) (9). Overall, few adverse events such as nausea, vomiting, dizziness, and dry mouth were reported; these were temporary, mild, and did not require additional medical care. As of this writing, there is one published report on WS-TCM delivery in a conventional US midwestern academic medical center (AMC) (10). In a single-arm study examining patient-reported outcomes of pain, anxiety, and depression in people engaging in WS-TCM with IVF, 67.1% of participants were ≥38 years, 79.5% were white, and 82.97% were non-Hispanic. In one private US IVF center, study participants who added WS-TCM to their fresh ETs had a higher proportion of diagnoses with diminished ovarian reserve, lower antral follicle count, and a previous failed cycle compared with those who did not add WS-TCM (8). A Chinese study found that before matching, people who received WS-TCM were older (32.1 ± 4.3 vs. 31.9 ± 3.9, *P* = .007), had a greater antral follicle count, had elevated testosterone levels, and underwent more gonadotropin-releasing hormone agonist long protocols than antagonist protocols compared with people who did not utilize WS-TCM (9).

Beyond basic demographics and reproductive-related history, a critical knowledge gap exists in understanding which patients engage in WS-TCM and what additional clinical characteristics and social drivers of health (SDoH) predict its use. Accordingly, the purpose of this study is to investigate sociodemographic and clinical variables associated with WS-TCM utilization alongside FET after IVF.

## Materials and methods

### Participants and design

This study is a retrospective electronic health record (EHR) review of all autologous FETs provided to women aged 21–42 receiving care at the University Hospitals (UH) Fertility Center between January 3, 2019 and March 31, 2024. FETs involving egg donors, embryo donors, or gestational carriers, and those missing live birth outcome data were excluded. Whole Systems Traditional Chinese Medicine exposure was defined as engaging in ≥1 WS-TCM treatment between 120 days before the FET date and 12 days post-transfer date as evidenced by a documented EHR note from a UH WS-TCM provider. This exposure period was chosen to allow WS-TCM treatment up to 4 months before and 12 days after ET. FETs among patients who only engaged in WS-TCM outside of the T-120 to T+12-day window were excluded.

### Setting and care delivery

The UH Fertility Center is a multidisciplinary center that facilitates close collaboration between clinical and laboratory teams and IHM practitioners. Whole Systems Traditional Chinese Medicine treatment delivery characteristics at the UH Fertility Center have been described previously (10). Briefly, care typically includes a regimen of 4–6 WS-TCM treatments delivered once weekly to prepare for ET and acupuncture on the day of transfer, with either one treatment before ET in an outpatient WS-TCM setting or one treatment before and one treatment after transfer on-site at the Fertility Center depending on patient preference. Patients have the option to follow up with weekly treatments after ET until the end of the first trimester of pregnancy. Appointments are scheduled for 90 minutes for an initial visit and 60 minutes for follow-up visits, with a total needle retention time of 25–30 minutes. Reimbursement is a mix of self-pay and commercial insurance. All practitioners are board certified by the National Certification Board for Acupuncture and Herbal Medicine (NCBAHM) and hold an acupuncture license from the State Medical Board of Ohio. Patients receive individualized acupuncture, counseling, and guidance with herbs, supplements, and lifestyle medicine.

### Ethics and permissions

This study was approved by the UH Cleveland Medical Center Institutional Review Board as a retrospective chart review (STUDY20250225) with a waiver of informed consent.

### Data collection

Primary FET event and reproductive health diagnosis data were extracted from a clinical database maintained by the UH Fertility Center. Additional sociodemographic, diagnostic (based on International Classification of Diseases-10 codes present and not discontinued before FET transfer date), and WS-TCM treatment data were extracted from the UH EHR using multiple structured query language scripts. These data included (1) number and timing of WS-TCM treatments; (2) sociodemographic information including age at the time of ET, race/ethnicity, marital status, and neighborhood-level median income derived from patients’ addresses; (3) clinical characteristics including body mass index (BMI) and having a previous medical history of a mental health or substance use disorder (MSUD) (24), headache/migraine diagnosis (G43–44, R51), musculoskeletal pain diagnosis (M50–54, M70–79, M99), or thyroid disorder (E00–E07); (4) whether preimplantation genetic testing (PGT) was performed in conjunction with the FET; (5) reproductive health history diagnoses (International Classification of Diseases-10 or documented in clinic database) including cervical dysplasia (N85, N87, N88), endometriosis (N80), polycystic ovary syndrome (PCOS) (E28.2), recurrent pregnancy loss (RPL) (N96), salpingitis/oophoritis/pelvic inflammatory disease (PID) (N70–71, N73, N76), noninflammatory disorders of the ovary, fallopian tube, and broad ligament (N83), polyps of the female genital tract (N84), other abnormal uterine/vaginal bleeding (N93), pain and other conditions associated with female genital organs and menstrual cycle (N94), history of hemorrhage in early pregnancy (O20) or antepartum (O46), history of high-risk pregnancy supervision (O09), and male fertility factor; and (6) reproductive health event data including prior full-term births and ART cycles.

### Data analysis

Model covariates were chosen based on their (1) clinical significance; (2) association with acupuncture, WS-TCM, or IHM exposure in prior studies (8, 18); and (3) prevalence within the study sample (i.e., n ≥ 20 in each cell in bivariate analysis). Given that multiple FETs were present per patient within the sample, we elected to randomly select one FET per patient using the “set.seed” and “sample_n” functions in R (25) to keep observations independent.

We examined all covariates for missingness before bivariate and regression analyses, with all missing data assumed to be missing at random. Of 1457 FETs in the original sample, 205 (14.1%) had missing data on neighborhood level-median income, and 37 (2.54%) had missing data on diagnoses documented in the EHR. We performed single imputation procedures using robust linear models and predictive mean matching from the “simputation” package (26). Descriptive statistics and bivariate analyses including Pearson’s Chi squared for categorical variables and Wilcoxon rank sum tests for continuous variables were conducted using the “tbl_summary” function (27).

We fit a logistic regression model using the “glm” function (25). Continuous variables were transformed to enable meaningful interpretations of coefficients (i.e., age in 10-year increments as well as scaled BMI and neighborhood median income). Race/ethnicity was coded as a binary non-Hispanic White variable given low representation of other racial and ethnic groups. Count variables (i.e., prior ART cycles and full-term births) were collapsed into ordinal categorical variables given small cell sizes. All analyses and plots were generated using R Version 4.5.1 (25) and RStudio Version 2025.09.2+418 (28).

## Results

### Sample characteristics

Figure 1 provides a flow chart of FET observation selection for this analysis. There were 1299 FETs among 799 patients meeting eligibility criteria. After filtering to one FET per patient (N = 799), 162 (20.3%) of these FETs were among patients utilizing WS-TCM. Patients (mean and SD age 34.93 ± 3.97 years) were mostly non-Hispanic White (78.6%) or Black/African American (8.1%) and married (83.9%). The median (interquartile range [IQR]) time between embryo frozen date and FET transfer date was 71 (30, 241.5) days. Table 1 summarizes sociodemographic and clinical covariates overall and by group.

**Figure 1:**
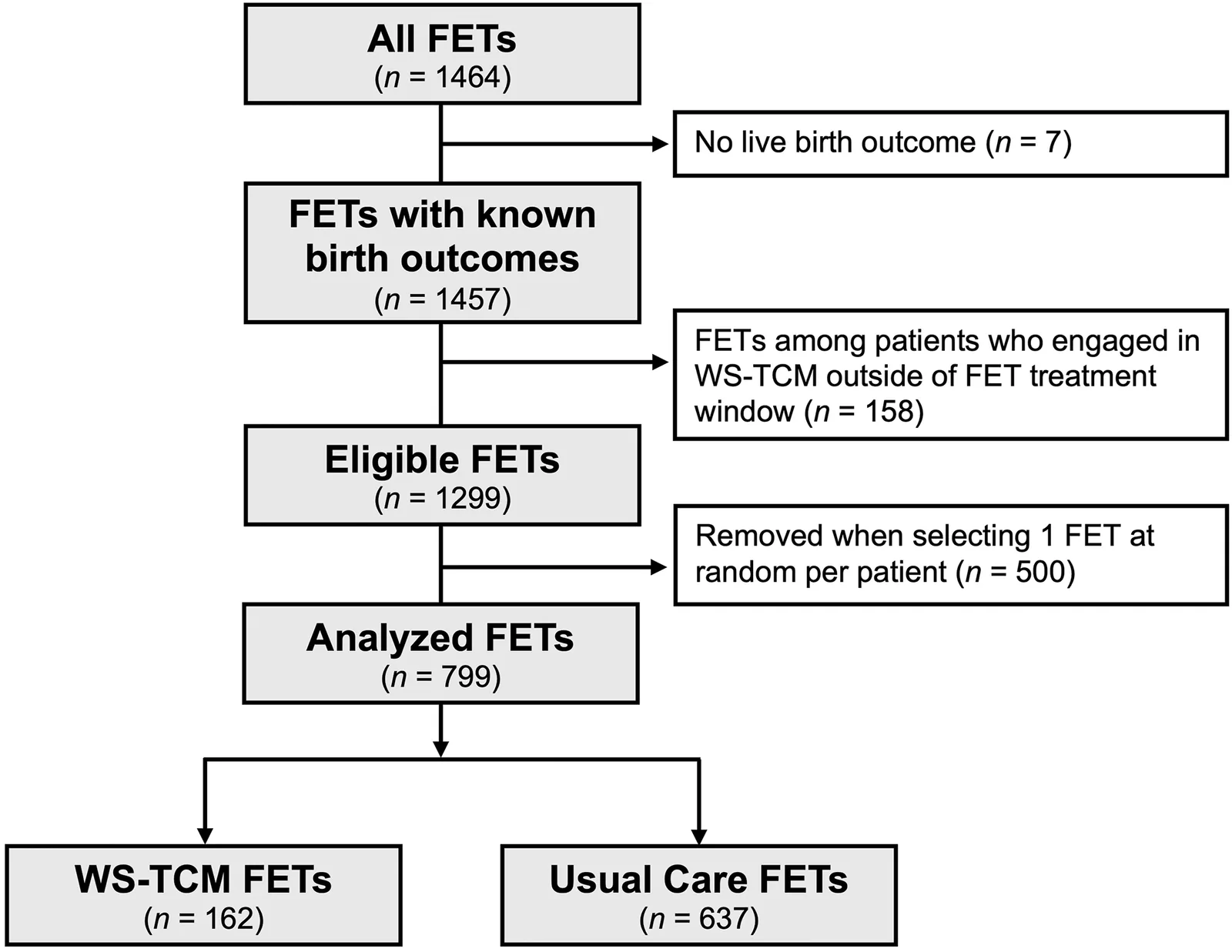
Flow chart of FET observation selection. FET = frozen embryo transfer; WS-TCM = Whole Systems Traditional Chinese Medicine.

**Table 1:** Bivariate analysis.

Variable	All N = 799	WS-TCM N = 162	Usual care N = 637	*P* value
Age (years)	34.93 ± 3.97	35.20 ± 3.64	34.86 ± 4.05	0.436
Race/ethnicity				0.416
NH White	628 (78.6%)	129 (79.6%)	499 (78.3%)	
NH Black/African American	65 (8.1%)	10 (6.2%)	55 (8.6%)	
Other race/Hispanic/Latino	63 (7.9%)	11 (6.8%)	52 (8.2%)	
Declined/Missing	43 (5.4%)	12 (7.4%)	31 (4.9%)	
Married	670 (83.9%)	142 (87.7%)	528 (82.9%)	0.141
Neighborhood median income	77,107 ± 25,195	83,025 ± 27,007	75,602 ± 24,508	0.002
Clinical characteristics				
BMI	28.25 ± 6.65	27.50 ± 6.36	28.45 ± 6.71	0.150
MSUD	223 (27.9%)	55 (34.0%)	168 (26.4%)	0.055
Migraine/headache	120 (15.0%)	35 (21.6%)	85 (13.3%)	0.009
Musculoskeletal pain	235 (29.4%)	60 (37.0%)	175 (27.5%)	0.017
Thyroid disorder	146 (18.3%)	40 (24.7%)	106 (16.6%)	0.018
PGT testing performed	220 (27.5%)	41 (25.3%)	179 (28.1%)	0.478
Reproductive health history				
Cervical dysplasia	113 (14.1%)	32 (19.8%)	81 (12.7%)	0.022
Endometriosis	121 (15.1%)	26 (16.0%)	95 (14.9%)	0.719
PCOS	179 (22.4%)	40 (24.7%)	139 (21.8%)	0.434
RPL	124 (15.5%)	42 (25.9%)	82 (12.9%)	<0.001
Salpingitis, oophoritis, or PID	224 (28.0%)	42 (25.9%)	182 (28.6%)	0.503
Non-inflammatory d/o ovary/fallopian tube	95 (11.9%)	26 (16.0%)	69 (10.8%)	0.067
Polyps of the female genital tract	136 (17.0%)	37 (22.8%)	99 (15.5%)	0.027
Other abnormal uterine/vaginal bleeding	88 (11.0%)	26 (16.0%)	62 (9.7%)	0.022
Pain associated with genital organs	124 (15.5%)	34 (21.0%)	90 (14.1%)	0.031
H/o hemorrhage in early pregnancy	69 (8.6%)	21 (13.0%)	48 (7.5%)	0.028
H/o high-risk pregnancy supervision	163 (20.4%)	28 (17.3%)	135 (21.2%)	0.270
Male fertility factor	283 (35.4%)	49 (30.2%)	234 (36.7%)	0.123
Prior full-term births				0.256
0	512 (64.1%)	110 (67.9%)	402 (63.1%)	
1+	287 (35.9%)	52 (32.1%)	235 (36.9%)	
Prior ART cycles				0.032
0–1	533 (66.7%)	95 (58.6%)	438 (68.8%)	
2	167 (20.9%)	39 (24.1%)	128 (20.1%)	
3+	99 (12.4%)	28 (17.3%)	71 (11.1%)	

*Note:* Continuous variables compared using Wilcoxon rank sum test. Binary and categorical variables compared using Pearson’s Chi-squared test. ART = assistive reproductive technology; BMI = body mass index; d/o = disorders of; H/o = history of; MSUD = mental health or substance use disorder; NH = non-Hispanic; PCOS = polycystic ovary syndrome; PID = pelvic inflammatory disease; PGT = preimplantation genetic testing; RPL = recurrent pregnancy loss; WS-TCM = Whole Systems Traditional Chinese Medicine.

### WS-TCM utilization

Among the 162 FETs featuring WS-TCM, a median (IQR) of 6.5 (2–11) treatments were provided per FET between T-120 and T+70 days relative to the transfer date. Of all FETs in this group, 116 (71.6%) included pre-transfer treatment, 144 (88.9%) included day-of-transfer treatment, and 57 (35.2%) included post-transfer treatment. Forty-one (25.3%) only featured day-of-transfer WS-TCM and 14 (8.6%) only included pre-transfer WS-TCM. Among the 116 FET that featured pre-transfer treatment, patients received a median of 5.5 (2–9) pre-transfer treatments with a median (IQR) start date of 68 (28–111) days before FET and a range of 1–120 days.

### Bivariate analysis

Table 1 presents the bivariate comparison of FETs by WS-TCM exposure. Compared with those not engaging in WS-TCM, WS-TCM utilizers resided in neighborhoods with higher median income ($83,025 vs. $75,602, *P* = .002). Their previous medical histories included higher rates of migraine/headache (21.6% vs. 13.3%, *P* = .009), musculoskeletal pain (37.0% vs. 27.5%, *P* = .017), thyroid disorders (24.7% vs. 16.6%, *P* = .018), cervical dysplasia (19.8% vs. 12.7%, *P* = .022), RPL (25.9% vs. 12.9%, *P*<.001), polyps of the female genital tract (22.8% vs. 15.5%, *P* = .027), other abnormal uterine/vaginal bleeding (16.0% vs. 9.7%, *P* = .022), pain and other conditions associated with female genital organs and the menstrual cycle (21.0% vs. 14.1%, *P* = .031), hemorrhage in early pregnancy or antepartum (13.0% vs. 7.5%, *P* = .028), and undergoing more ART cycles (undergoing ≥3 17.3% vs. 11.1%, *P* = .032). There was also a higher prevalence of MSUD within the WS-TCM group (34.0% vs. 26.4%), but this was not statistically detectable (*P* = .055).

### Predictors of WS-TCM engagement

Table 2 and Figure 2 summarize aOR and 95% CI from the logistic regression model. Covariates associated with higher odds (aOR [95% CI]) of WS-TCM utilization included higher neighborhood median income (1.36 [1.13, 1.64] per SD increase), RPL (2.15 [1.33, 3.47]), and undergoing ≥3 vs. 0–1 ART cycles (1.78 [1.02, 3.06]). Higher BMI (0.79 [0.64, 0.97] per SD increase), undergoing PGT (0.61 [0.39, 0.94]), and having a history of salpingitis/oophoritis/PID (0.59 [0.37, 0.92]) or high-risk pregnancy supervision (0.53 [0.30, 0.93]) were associated with lower odds of WS-TCM utilization.

**Table 2:** Logistic regression model coefficients.

Fixed effect	aOR	95% CI	*P* value
Age +10 years	1.17	0.70–1.97	0.542
NH White	0.88	0.55–1.44	0.614
Married	1.35	0.79–2.41	0.291
Neighborhood median income +1 SD	1.36	1.13–1.64	0.001
BMI +1 SD	0.79	0.64–0.97	0.026
MSUD dx	1.32	0.86–2.00	0.195
Migraine/HA	1.61	0.97–2.63	0.059
MSK pain	1.22	0.80–1.85	0.355
Thyroid disorder	1.49	0.94–2.34	0.086
PGT testing	0.61	0.39–0.94	0.027
Cervical dysplasia	1.29	0.76–2.17	0.334
Endometriosis	0.94	0.53–1.61	0.821
PCOS	1.26	0.79–2.01	0.324
RPL	2.15	1.33–3.47	0.002
Salpingitis/oophoritis/PID	0.59	0.37–0.92	0.024
Noninflammatory d/o ovary/fallopian	1.23	0.68–2.17	0.483
Polyp of female genital tract	1.58	0.96–2.55	0.067
Other abnormal uterine/vaginal bleeding	1.35	0.75–2.37	0.304
Pain associated with female genital organs	1.45	0.86–2.39	0.155
Hemorrhage before pregnancy	1.69	0.89–3.13	0.103
Supervision of hi-risk pregnancy	0.53	0.30–0.93	0.029
Male infertility factor	0.86	0.57–1.28	0.459
≥1 prior full-term birth	0.96	0.61–1.48	0.844
2 v 0–1 prior ART cycles	1.33	0.83–2.08	0.227
3+ v 0–1 prior ART cycles	1.78	1.02–3.06	0.039

*Note:* aOR = adjusted odds radio; ART = assistive reproductive technology; BMI = body mass index; CI = confidence interval; d/o = disorders of; HA = headache; MSK = musculoskeletal; MSUD = mental health or substance use disorder; NH = non-Hispanic; PCOS = polycystic ovary syndrome; PID = pelvic inflammatory disease; PGT = preimplantation genetic testing; RPL = recurrent pregnancy loss; SD = standard deviation.

**Figure 2:**
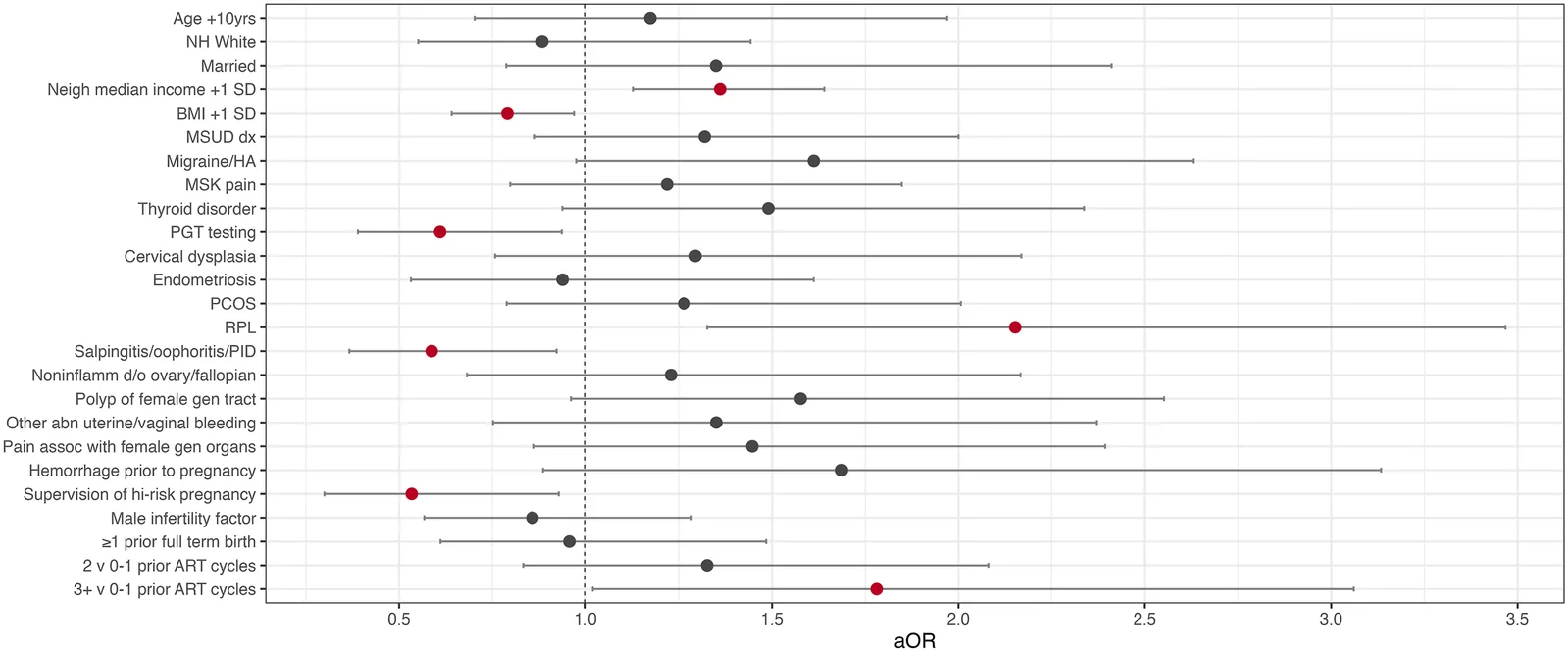
Adjusted odds ratios from the logistic regression model. Dots represent point estimates for adjusted odds ratios, and error bars represent 95% confidence intervals. Red dots are covariates where the confidence interval did not include 1. ART = assistive reproductive technology; BMI = body mass index; d/o = disorders of; gen = genital; HA = headache; MSK = musculoskeletal; MSUD = mental health or substance use disorder; Neigh = neighborhood; NH = non-Hispanic; PCOS = polycystic ovary syndrome; PID = pelvic inflammatory disease; PGT = preimplantation genetic testing; RPL = recurrent pregnancy loss.

## Discussion

This study identified sociodemographic and clinical variables associated with WS-TCM utilization alongside autologous FET cycles. In general, our sample revealed that people using WS-TCM had greater medical complexity, a higher prevalence of pain-related diagnoses, reproductive-related pathologies, and RPL, and had previously completed more ART cycles than those who did not utilize WS-TCM. Migraine/headaches, musculoskeletal pain, endometriosis-related pelvic pain, and genital pain diagnoses were more prevalent among WS-TCM users. This is unsurprising as pain-related acupuncture referrals are common and evidence-based (29, 30). Lu et al. (10) also reported that pain was commonly cited as a concern for people utilizing WS-TCM. Health histories among individuals utilizing WS-TCM also included more reproductive-related diagnoses like cervical dysplasia (cervical intraepithelial neoplasia), thyroid pathologies, polyps of the reproductive tract, and abnormal menstrual bleeding patterns. Patients utilizing WS-TCM were also more likely to have a history of RPL and to have undergone a higher number of previous ART treatments. Although acupuncture is not recommended as a first-line adjuvant therapy in American Society for Reproductive Medicine IVF guidelines (31), people with RPL or multiple failed cycles may seek additional therapies despite limited evidence (15, 32, 33). Further, patients undergoing IVF may feel compelled to leave no opportunity unexplored to potentially optimize their chances of successful pregnancy (34, 35, 36).

The stress of family building with ART is well documented (37, 38, 39, 40, 41). Individuals undergoing IVF often report elevated distress (42), and enduring care can be burdensome due to cost (32, 43), psychological burden (38, 44, 45, 46), physical impacts (e.g., invasive procedures, self-administered injections, and discomfort), negative cycle outcomes (e.g., cycle delays, cancellation, no pregnancy, or miscarriage) (47), and communication challenges with healthcare professionals (34). Although cost is a considerable stressor and even barrier to care, Domar et al. (47) found that stress was the primary reason participants reported stopping IVF despite not delivering a baby (33, 34, 35, 36). Prior studies have also demonstrated acupuncture’s benefits for improving resilience (35, 36) and reducing stress (19, 35, 36, 48, 49, 50, 51).

This study demonstrated that individuals utilizing WS-TCM may have more financial means to pursue other adjuvant treatments out-of-pocket. They resided in neighborhoods with higher median income compared with those not utilizing WS-TCM. This is consistent with prior National Health Interview Survey studies indicating people who used IHM approaches had higher incomes (18). In vitro fertilization care in the United States is still largely provided in the private sector, with limited insurance coverage and substantial treatment costs. The average IVF cycle can range between $12,400 (52) without medications and $60,000 (43) in out-of-pocket costs for people without insurance coverage. Additional cost burden associated with acupuncture treatment is possible as health insurance reimbursement may not be accessible, and coverage can be limited to pain and migraine diagnoses, excluding any infertility-related diagnosis codes. At our center, insurance is accepted for acupuncture, and at least one employer-based payer covers acupuncture without restriction to diagnosis code.

In this study, the median number of WS-TCM treatments was 5.5 beginning more than 2 months before the FET; this number of treatments is fewer than has been reported in other studies. Hullender Rubin et al. (8) reported a mean ± SD of total WS-TCM treatments of 12.0 ± 12.4 alongside fresh IVF cycles. Song et al. (9) reported WS-TCM was initiated in the cycle preceding FET with sessions every 2 days until ET, although the total number of visits was not reported. The ideal dosage and timing of treatments to best support IVF, whether during fresh stimulation and transfer or frozen transfer, remain unclear.

Of those who did not engage with WS-TCM, our study demonstrated that they (1) were more likely to have PGT-tested embryos, (2) were larger bodied (elevated BMI), and (3) had a higher prevalence of inflammatory disorders of the reproductive tract and high-risk pregnancy supervision documented in their previous medical history. Preimplantation genetic testing may provide a sense of security and increase cost burden (53), potentially restricting financial resources for additional therapies. Patients undergoing IVF who add PGT-A to their treatment cycles report confidence in the scientific advancement and anticipate positive effects on their IVF outcomes (53). Patients’ choice to utilize PGT may be driven by the potential to decrease the likelihood of miscarriage or termination. However, patients may also consider increased financial costs (54), emotional burdens, and heightened uncertainty throughout the process (55).

WS-TCM’s influence on IVF outcomes with PGT-tested embryos is unclear. In a retrospective study at a private, for-profit, IVF center, ≥3 WS-TCM sessions (n = 61) were associated with improved birth outcomes in FETs with genetically untested autologous embryos compared with day-of-ET acupuncture (n = 33, 59.0% vs. 33.3%, *P* = .02) (56). More live births occurred in the WS-TCM group compared with FET alone (n = 181), but this difference was not significant (59.0% vs. 50.0%, *P =* .07). In a second retrospective study of PGT-tested ETs from the same center, receiving ≥3 WS-TCM sessions (n = 359) was associated with more FET live births than day-of-ET acupuncture (n = 150, 64.6% vs. 55.3%, *P =* .05), suggesting that dose and timing of treatment may be important variables to consider for future investigations. In this same study, there was no meaningful difference in live births between the FET-alone (n = 526) group and WS-TCM (n = 359) group (67.7% vs. 64.6%, *P =* .35) (57).

People with elevated BMI were less likely to utilize WS-TCM. Although higher BMI is more prevalent across underrepresented minorities, our cohort was largely white/non-Hispanic (58, 59). Referral to a weight management program may have been prioritized in people with BMI ≥ 40 over referral for WS-TCM. Still, not adding another provider may have been a decision based in avoidance due to perceived weight bias or stigma (60). Larger-bodied people may delay or avoid care for this reason. Additionally, in our cohort, people with a medical history that included a high-risk pregnancy, oophoritis, salpingitis, or PID were less likely to utilize WS-TCM. Fear of a new therapy may also be a perceived risk despite the established safety of acupuncture (61, 62) and WS-TCM during ART (8, 9, 20, 63) and pregnancy (64) when provided by trained providers. Future mixed-methods studies could explore psychological factors and perceptions influencing patients’ decisions to engage in WS-TCM.

Unexpectedly, mental health diagnoses were not associated with WS-TCM use in our sample. Although there was a higher prevalence of mental health disorders in the WS-TCM group (34.0% vs. 26.4%), it was not significant (*P* = .055) and likely underpowered to detect differences. In a meta-analysis of eight trials, acupuncture therapy reduced IVF-related anxiety (SMD −0.21, 95% CI −0.39, −0.04) (65). Another meta-analysis was unable to analyze the two identified trials that investigated acupuncture’s impact on IVF-related depression (66). Future investigations should account for this important comorbidity.

Findings from this study inform the selection of covariates that should be accounted for in future observational studies. Most acupuncture studies to date have investigated day-of-ET treatment during fresh IVF cycles, with primary outcomes focused on pregnancy outcomes, anxiety, and stress (20, 21, 67, 68, 69, 70). There remains a significant knowledge gap in understanding WS-TCM’s real-world effects on FET birth outcomes, especially in the United States. In FET cycles with autologous embryos with (57) or without (56) genetic testing, approximately four WS-TCM sessions before transfer plus two acupuncture sessions on the day of ET were associated with more live births compared with day of ET acupuncture only, but there was no association when compared with usual care in either cycle type. Phillippi et al. (71) reported that individualized acupuncture on the day of ET in autologous FET cycles was associated with 55% more live births and 42% fewer biochemical pregnancies compared with no acupuncture. Our study suggests that future observational studies should account for covariates such as pain-related diagnosis history, RPL, SDoH, and reproductive health history when applying propensity-score matching techniques.

There are several strengths of this study. This is the first investigation to consider pain-related, mental health, and SDoH variables influencing WS-TCM utilization alongside IVF in a US AMC. This real-world dataset reflects how patients undergoing IVF might engage with WS-TCM during an FET treatment cycle. When provided in the real world, acupuncture is often deployed as part of an individualized, multimodal intervention like WS-TCM; therefore, WS-TCM is more likely the actual intervention one receives.

Limitations of our study include a small sample within a single midwestern AMC. We were unable to acquire data on education level and self-reported psychological distress, because these items were not formally collected in our EHR. We also cannot account for exposure to WS-TCM outside of our hospital system, which could mean we were unable to capture other variables of interest. In addition, specific variables we observed in our cohort may be over- or underrepresented in our sample. We used binary disease classifications that did not account for disease severity or etiology. The cross-sectional design with one FET per person does not adequately inform factors related to WS-TCM engagement after 1 or more FETs without WS-TCM. Finally, income data included in our data were imputed based on neighborhood rather than individual level. Area-level socioeconomic indicators are frequently used when individual-level data are unavailable. However, these measures often misclassify individuals’ socioeconomic status (72, 73).

## Conclusions

Within this cohort, multiple pain- and reproductive health-related diagnoses were more prevalent among individuals utilizing WS-TCM. These findings inform factors that may influence clinicians’ and patients’ decisions to refer or pursue WS-TCM (e.g., migraine, musculoskeletal pain, RPL) and covariates that should be accounted for in future observational studies of WS-TCM’s effectiveness on outcomes including rates of clinical pregnancy and live birth.

## Declaration of Interests

S.N.R-.M. report funding from Elisabeth Severance Prentiss Foundation for the submitted work. L.H.R. report funding from Elisabeth Severance Prentiss Foundation for the submitted work. S.T.K. report funding from Elisabeth Severance Prentiss Foundation for the submitted work. L.D.J. report funding from Elisabeth Severance Prentiss Foundation for the submitted work. R.F. report funding from Elisabeth Severance Prentiss Foundation for the submitted work. C.K. report funding from Elisabeth Severance Prentiss Foundation for the submitted work.
